# Effects of Cu-Coated SiC Content on Microstructure and Properties of Laser Cladding SiC_p_/Al–Si Composite Coatings

**DOI:** 10.3390/ma12091537

**Published:** 2019-05-10

**Authors:** Yang Liu, Guodong Li, Wenting Jiang

**Affiliations:** State Key Laboratory of Powder Metallurgy, Central South University; Changsha 410012, Hunan, China; 163311060@csu.edu.cn (Y.L.); jwtwendy2013@163.com (W.J.)

**Keywords:** laser cladding layer, laser processing, electroless copper, microhardness

## Abstract

SiC particles (SiC_p_)-reinforced Al–Si matrix composite coatings were synthesized on 4032 aluminum alloy by laser cladding using powder mixtures of Al-20 wt.% Si alloy and electroless copper-plated SiC particles (SiC_p-Cu_). The effects of SiC_p-Cu_ content on microstructure, phase composition, and microhardness of the SiC_p_/Al–Si laser cladding layer (LCL) were investigated systematically. The results showed that the microstructure of SiC_p-Cu_/Al–Si LCL was mainly composed of undissolved SiC_p_, lump-like primary Si, lump-like Al_2_Cu, plate-like Al_4_SiC_4_, and Al–Si–Cu ternary eutectic. In addition, the eutectic microstructure became finer with the increasing of SiC_p-Cu_ content. The average microhardness of the LCL increased with the increasing of SiC_p-Cu_ content. When SiC_p-Cu_ content was 50 wt.%, the average microhardness of the LCL reached 508 HV_0.05_, which was about 3.5 times larger than that of the substrate. The LCL reinforced with a SiC_p-Cu_ content of 30 wt.% exhibits the best wear resistance.

## 1. Introduction

Aluminum alloys are extensively applied in the automotive industry and aircraft and other fields due to their reduced density, light weight, and high specific values of stiffness and strength. Nevertheless, the use of aluminum alloys for a wider range of application is limited due to their low surface hardness and poor wear resistance [1,2,3,4]. Therefore, it is necessary to improve surface properties and mechanical properties and prolong the service life of the parts made from aluminum alloys [5,6,7]. In order to improve surface properties of aluminum alloys, various attempts have been made, such as electroplating [8], electroless plating [9], thermal spraying [10], anodizing [11], and microarc oxidation [12]. However, the disadvantages of pollution of the environment and weak adhesion between the substrate and the coating still exist for these methods mentioned above, resulting in the difficulty of meeting the requirements under severe conditions. Compared with conventional surface treatment methods, the laser cladding process has the advantages of low clad dilution, rapid heating and cooling, small heat-affected zone, and good adaptability of surface properties [13,14,15,16]. 

Considerable research studies have been carried out to examine laser treatment of aluminum alloys. Sun et al. [17] fabricated composite coatings of Al–Si alloy reinforced with SiC particles on AlSi12 substrate; the microstructure and microhardness of the coatings were investigated, and the results showed that the coatings had much higher microhardness than that of the substrate, and the coatings were divided into two sublayers; the upper layer was composed of Al–Si eutectic, acicular primary Si, α-Al dendrites, and a little SiC_p_, while the bottom layer consisted of α-Al dendrites, Al–Si eutectic, and a large amount of SiC_p_. The oxidation effects during the laser treatment of aluminum coated with SiC_p_/Al composite coating was studied by Hegge et al. [18], and the authors found that inert gas stream is not always enough to sufficiently prohibit contact between the air and the melt. Anandkumar et al. [19] studied the influence of the laser cladding process on the microstructure and abrasive wear resistance of SiC_p_/Al–Si composite coating; they observed that the microstructure and properties of the SiC_p_/Al–Si laser cladding layer (LCL) depends strongly on the processing parameters, especially power density and interaction time. The influence of addition of alloy elements on microstructure and microhardness of the SiC_p_/Al–Si LCL was investigated by Riquelme et al. [13]. The result indicated that the addition of Si or Ti particles to the composite coating is an effective method to avoid the formation of Al_4_C_3_. However, there is no research on the effect of electroless copper plating on SiC_p_ on the properties of SiC_p_/Al–Si LCL.

In the process of laser cladding, SiC_p_ tends to react with molten aluminum, leading to the formation of Al_4_C_3_ and Al_4_SiC_4_ and so on during solidification depending on temperature [20]. Between 667 °C and 1347 °C, reaction (1) takes place and produces Al_4_C_3_. When the temperature exceeds 1347 °C, reaction (2) takes place. When the temperature reaches 1927 °C, Al_8_SiC_7_ will be formed [21].

4Al_(l)_ + 3SiC_(s)_ → Al_4_C_3(s)_ + 3Si, (1)

4Al_(l)_ + 4SiC_(s)_ → Al_4_SiC_4(s)_ + 3Si, (2)

The hardness of Al_4_SiC_4_ is as high as 1200 HV and its brittleness is low. In addition, it is chemically inert in humid environments, so Al_4_SiC_4_ is a favorable reinforcement phase [22,23]. In the process of preparing SiC_p_/Al–Si LCL, the Al_4_SiC_4_ phase is desired. The poor wettability between Al and SiC_p_ will adversely reduce the reaction rate between Al and SiC_p_ during the laser cladding process and the bonding strength between Al matrix and SiC_p_. 

In this work, the wettability between SiC_p_ and Al alloy is expected to be improved by electroless copper plating on the surface of SiC_p_. SiC_p_/Al–Si coatings have been deposited on 4032 aluminum alloy by the laser cladding process. The effects of the SiC_p-Cu_ content on microstructure and properties of the LCL have been investigated for the first time. The purpose of this paper is to provide a technical way to improve surface properties of aluminum alloys.

## 2. Materials and Methods

### 2.1. Substrate and Cladding Material

The 4032 aluminum alloy was used as substrate for laser cladding with a dimension of 50 mm × 18 mm × 4 mm. The surface of the substrate was ground with abrasive paper and cleaned with alcohol before laser cladding. 

The cladding material was a mechanical mixture of AlSi20 aluminum alloy and SiC_p_ (including SiC_p_ or SiC_p-Cu_) powders. The AlSi20 aluminum alloy powder used had a particle size of 50–100 μm and the SiC_p_ had a particle size of 10–20 μm. The SiC_p_ and AlSi20 powders were mixed in different compositions as shown in Table 1. The mixed powders were placed onto the surface of aluminum alloy with gum water as binders and dried at 80 °C for 6 h. The thickness of the precoated layer was approximately 0.5 mm.

### 2.2. Electroless Plating of SiC Particles

**Figure 1:**
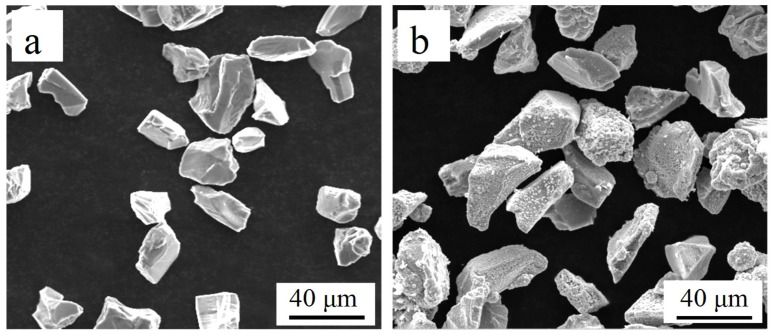
The SEM image of (**a**) uncoated SiC_p_, (**b**) SiC_p-Cu_.

Before electroless plating, surface treatment was carried out on the SiC_p_. According to previous studies, pretreatment can be conducted by the traditional three-step method (coarsening, sensitization, and activation) [24,25]. After pretreatment mentioned above, electroless plating was conducted in a copper electroless bath. The composition and operating conditions of pretreatment solutions and electroless copper plating bath are displayed in Table 2. Lastly, the Cu-coated SiC_p_ was washed with deionized water three times and dried under room temperature. Figure 1 shows the SEM images of SiC_p_ and SiC_p-Cu_.

### 2.3. Laser Cladding Experiment

Laser cladding was performed by using the CY-WL600G type Nd-YAG pulsed laser with a wavelength of 1.06 μm. Based on the systematic experiments done previously, the laser cladding process parameters used in this study were 800 W for laser beam power, 4 mm/s for laser scan speed, and 0.2 mm for laser beam diameter. There was a 50% overlap between two adjacent laser tracks. The thickness of LCLs obtained was about 0.45 mm. After laser processing, samples were cut for cross-section and polished with abrasive paper.

The microstructures of the LCL were analyzed by 10XB-PC optical microscope (OM) from Shanghai optical instrument factory and QUATA 250 FEG series field emission scanning electron microscope (SEM) from FEI. Semiquantitative analysis of element distribution was carried out by energy dispersive spectrometer (EDS), which was equipped with SEM. The LCL phase was tested by a DX2700 diffraction analysis system (XRD) from Shanghai Precision Instruments with Cu Kα radiation and XRD patterns were taken at 2*θ* angles from 15° to 85° at a scanning rate of 4°/min. Also, a BUEHLER5104 microhardness tester from German Buehler was used to obtain Vickers microhardness profiles along samples cross-section up to 750 μm using a load of 50 g for 10 s. For each sample, the microhardness measurements were repeated at five locations at the center and edges of the samples. The given average values of microhardness were average values taking from all measurement points on LCLs. Wear experiments were carried out using a PRN01-04882A pin-on-disk-type tribometer from Swiss CSM Company under dry-sliding conditions. The diameter of pin samples was 3 mm. The ring of the wear couple was made of diamond. The wear conditions were given as 1.4 MPa, 0.4 m·s^−1^ sliding speed, and 250 m sliding distance. Wear was characterized using the mass loss of the samples and the observation of the wear scars.

## 3. Results and Discussion

### 3.1. Phase Analysis

**Figure 2:**
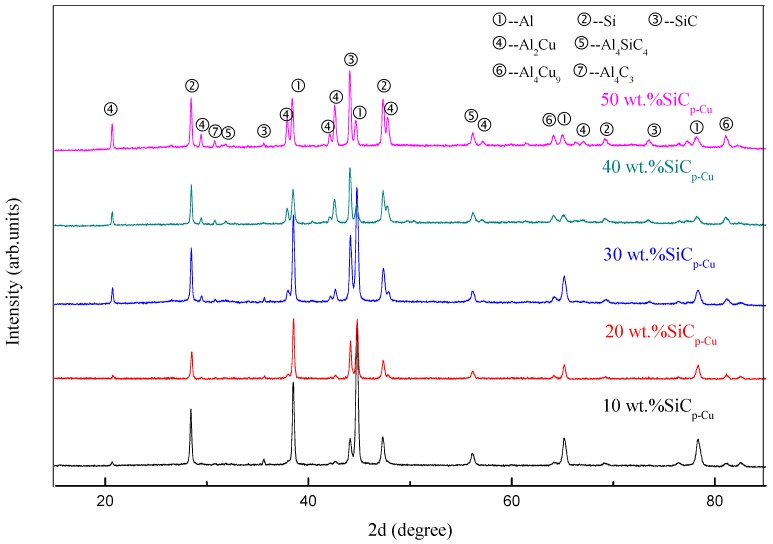
XRD results of laser cladding layer with different SiC_p-Cu_ content.

The XRD patterns of SiC_p-Cu_/Al–Si coatings with different SiC_p-Cu_ content are shown in Figure 2. As can be seen, in addition to Al and Si phases, great amounts of SiC_p_, Al_2_Cu, Al_4_Cu_9,_ Al_4_SiC_4_, and Al_4_C_3_ were found in the LCLs. The SiC_p_ was the additive and Al_2_Cu, Al_4_Cu_9_, Al_4_SiC_4_, and Al_4_C_3_ were in situ formed novel phases. Furthermore, with increasing SiC_p-Cu_ content, more and more Al_2_Cu, Al_4_Cu_9_, Al_4_SiC_4_, and Al_4_C_3_ compounds formed within the LCL and their diffraction peaks became obvious.

Table 3 describes the variation trend of 2*θ* values and intensities of Al peaks in LCLs with different SiC_p-Cu_ content. At the same time, the 2*θ* values of the standard diffraction peak of Al are also listed. It can be seen that when the SiC_p-Cu_ content is less than 50%, the 2*θ* values of Al diffraction peaks in the LCLs increase with increasing SiC_p-Cu_ content, and all of them are larger than the standard 2*θ* values. When the SiC_p-Cu_ content is 50 wt.%, the 2*θ* value of Al diffraction peak is smaller than the standard 2*θ* value. According to Bragg’s law [26], 2d sin *θ* = n*λ* (n = 1, 2, 3, …), the larger 2*θ* values indicate the smaller interplanar spacing of the corresponding crystal planes. It implies that the lattice deformation of the aluminum was caused by the high cooling rate and the huge residual stress during the laser cladding process.

As can be seen from Table 3, the intensity of XRD diffraction peaks of Al decreases with the increasing of SiC_p-Cu_ content in cladding materials. Meanwhile, the half high width of the XRD diffraction peaks (FWHM) of Al increases. On the basis of the Scherrer formula [26], $D=\frac{K\lambda }{B\cos\theta }$ (where *K* is the Scherrer constant, *D* is the average thickness of the grain perpendicular to the direction of the crystal plane, *B* is the half high width of the diffraction peak of the measured sample, *θ* represents the diffraction angle, and *λ* is the X-ray wavelength), the increase of FWHM of Al indicates that the grain size of Al matrix decreased. It indicates that the crystal structure of the SiC_p-Cu_/Al-Si composite coating produced by laser cladding process was significantly refined. When the content of SiC_p-Cu_ was 40 wt.%, the FWHM of Al was the largest, which means that the grain refinement was the most significant.

### 3.2. Microstructural Analysis

**Figure 3:**
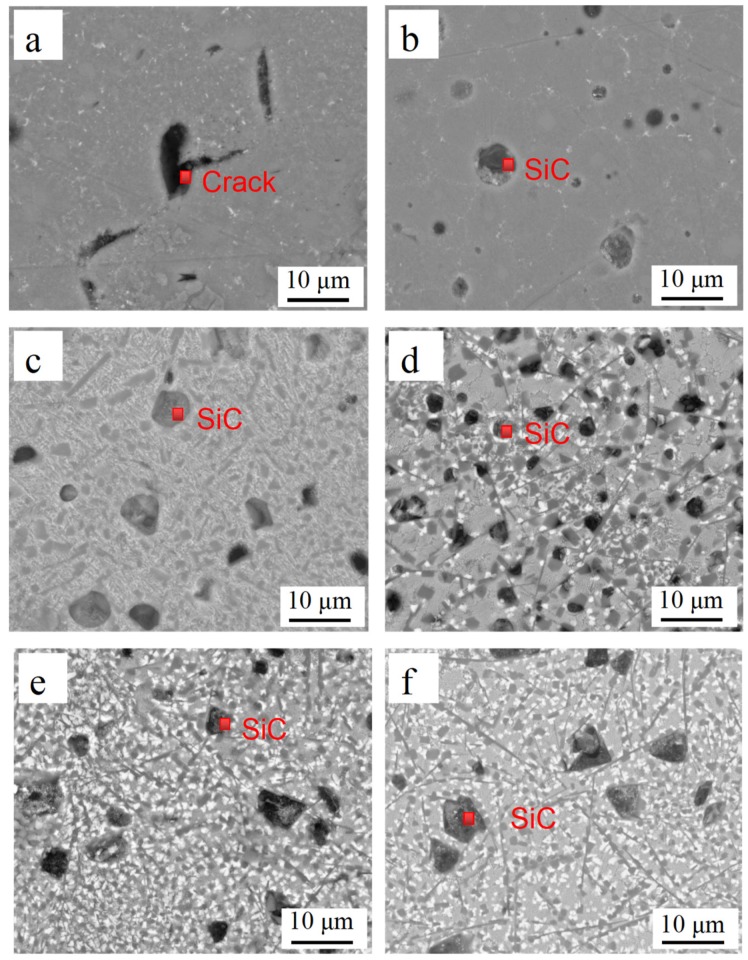
SEM images of laser cladding layer with different content of SiC_p-Cu_ (**a**) 0 wt.%; (**b**) 10 wt.%; (**c**) 20 wt.%; (**d**) 30 wt.%; (**e**) 40 wt.%; (**f**) 50 wt.%.

Figure 3 shows the SEM images of the cross-section of the LCLs with different SiC_p-Cu_ content. SiC_p-Cu_ with different sizes and shapes within the LCLs is observed. The SEM micrograph of the LCL without SiC_p-Cu_ is shown in Figure 3a; it can be seen from the figure that the grain size is coarser than that in the coating reinforced with SiC_p-Cu_ (Figure 3b–f). It can be seen from Figure 3a that there are cracks in the coating, and the existence of the cracks will have a negative impact on the properties of the coating. As seen in Figure 3b–f, the eutectic microstructures of the laser cladding layer become finer with increasing SiC_p-Cu_ content due to the LCLs absorbing rapid heating and cooling during the laser cladding process and possess fast solidification [27]. In addition, these solidification rates increase with the increasing of thermal conductivity and the SiC_p-Cu_ content, and as a result, thermal conductivity of SiC_p_ (259 Wm^−1^K^−1^) [28] and copper (401 Wm^−1^K^−1^) is higher than that of aluminum (237 Wm^−1^K^−1^) [29]. 

**Figure 4:**
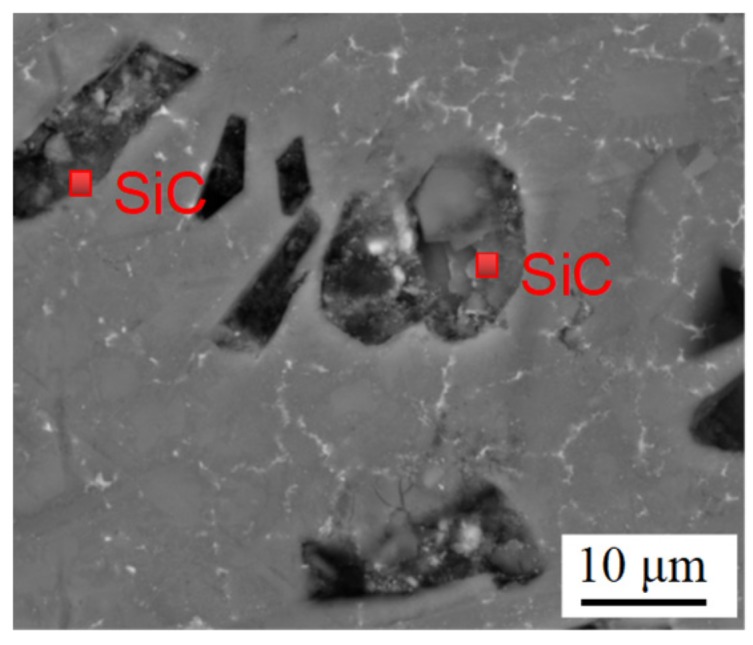
SEM images of a laser cladding layer with a SiC_p_ content of 20 wt.%.

The SEM micrographs of LCL with 20 wt.% of SiC_p-Cu_ and 20 wt.% of SiC_p_ are demonstrated in Figure 3c and Figure 4, respectively. The SiC_p_ remained almost unmelted and was still in an irregular polygonal shape as is shown in Figure 4. Conversely, the most SiC_p-Cu_ was oval-shaped and had a smaller size than that of the SiC_p-Cu_ originally used. It is indicated that the wettability of aluminum melt and SiC_p_ can be improved by electroless copper plating process. SiC_p-Cu_ is more likely to react with molten aluminum during the laser cladding process. It can be seen from Figure 3 that some SiC_p_ were also in an irregular polygonal shape. This is because not all SiC_p_ were coated entirely with copper during electroless plating as shown in Figure 1.

**Figure 5:**
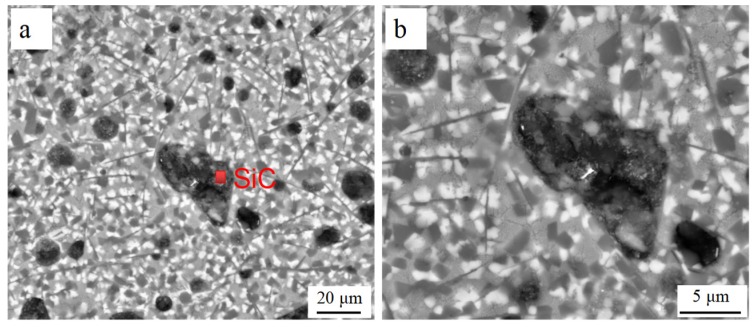
Microstructures of a laser cladding layer with a SiC_p-Cu_ content of 50 wt.%.

The microstructure of the LCL with SiC_p-Cu_ content of 50 wt.% at a higher magnification is shown in Figure 5. It is clear that the microstructure of the LCL was mainly composed of undissolved SiC_p_ and dark gray lump-like crystals which were distributed on the ternary eutectic of Al–Si–Cu. 

The Si content of AlSi20 alloy powders used in cladding materials is 20 wt.%. At the same time, SiC_p-Cu_ reacts with molten aluminum and forms Al_4_SiC_4_ and Si during the laser cladding process. The Si is dissolved in AlSi20, increasing its Si percentage, so that the content of Si in the molten aluminum could exceed the eutectic point. On the basis of the Al–Si binary phase diagram, the hypereutectic Al–Si matrix microstructure consisted of Al–Si eutectic and acicular primary Si crystals [17].

After electroless copper plating of SiC_p_, the weight gain percentage of SiC_p_ is close to 100%, so the cladding material with SiC_p-Cu_ content of 50 wt.% is comprised of 50 wt.% of Al–Si powder, 25 wt.% of SiC_p_, and 25 wt.% of Cu. The Al–Cu–Si ternary eutectic alloy is mainly composed of primary crystal Si, Al + Al_2_Cu binary eutectic, and Al + Si + Al_2_Cu ternary eutectic composition due to the high cooling rate (in the range of 10^3^–10^8^ K/s during the laser cladding process) [30,31,32]. 

**Figure 6:**
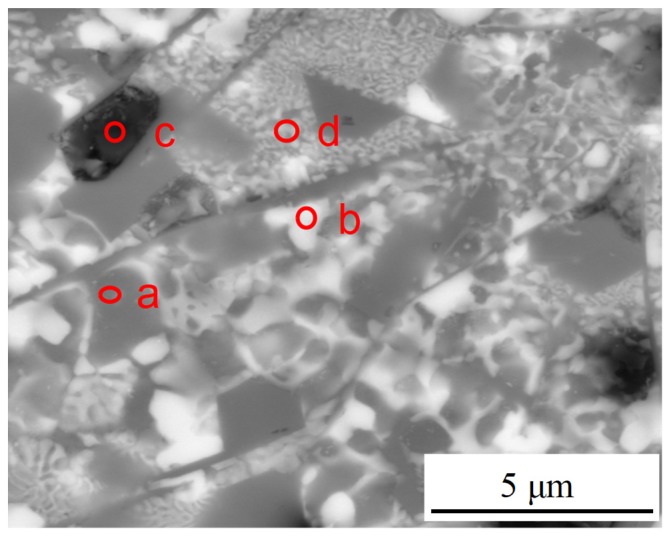
Microstructure of a laser cladding layer with a SiC_p-Cu_ content of 50 wt.%.

**Figure 7:**
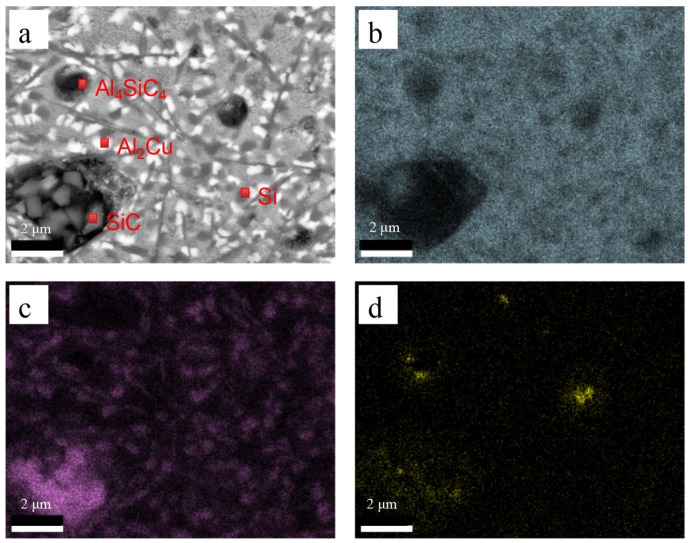
Distribution of aluminium (**b**), silicon (**c**), and carbon (**d**) of microstructure of the laser cladding layer with a SiC_p-Cu_ content of 50 wt.% (**a**).

EDS analysis was performed to examine the exact composition of LCL with SiC_p-Cu_ content of 50 wt.%. Results of EDS analysis conducted on points a–d in Figure 6 are then summarized in Table 4. Combining with the XRD phase analysis (Figure 2), it was reasonable to consider that the white lump-like crystals (point b) were Al_2_Cu, the black plate-like crystals (point c) were Al_4_SiC_4_, and the light gray region (point d) was Al + Al_2_Cu binary eutectic. The dark gray lump-like crystals (point a) in Figure 6 may be Si. Figure 7a shows the SEM image of the laser cladding layer. Figure 7b–d show the elemental maps of the LCL corresponding to the distribution of Al, Si, and C, respectively. According to that, the dark gray lump-like crystals (point a) in Figure 6 and acicular crystals can be recognized as Si phase. In summary, the microstructure of SiC_p-Cu_/Al–Si LCL mainly comprises undissolved SiC_p_, lump-like primary Si, lump-like Al_2_Cu, plate-like Al_4_SiC_4,_ and Al–Si–Cu ternary eutectic. 

**Figure 8:**
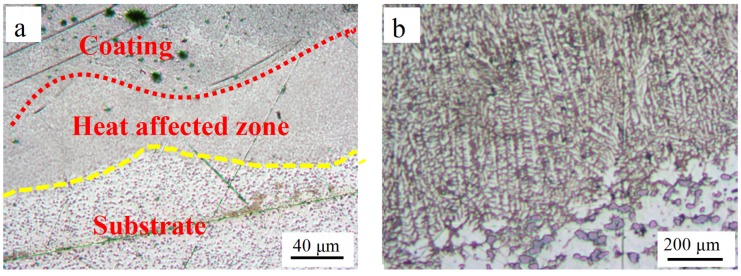
Interface morphologies of SiC_p-Cu_/Al–Si laser cladding before etching (**a**) and after etching (**b**).

Figure 8 shows the interface between the LCL and the substrate. (The samples were cut perpendicular to the LCL direction and polished. The images were taken in the center of the LCL region.) OM images of the coating before etching are presented in Figure 8a. The curved edges of the interface between the coating and the substrate caused by the laser beam center had a higher temperature than that of the edge region. After etching the surface of the LCL using the Keller’s reagent, the OM images obtained are shown in Figure 8b. It can be seen that there are many orientated growth dendrites between the substrate and the coating, and the growth direction is substantially perpendicular to the substrate. Moreover, the LCLs show good metallurgical bonding to the substrate due to the orientated growth dendrites which are intergrown with the substrate.

### 3.3. Microhardness

**Figure 9:**
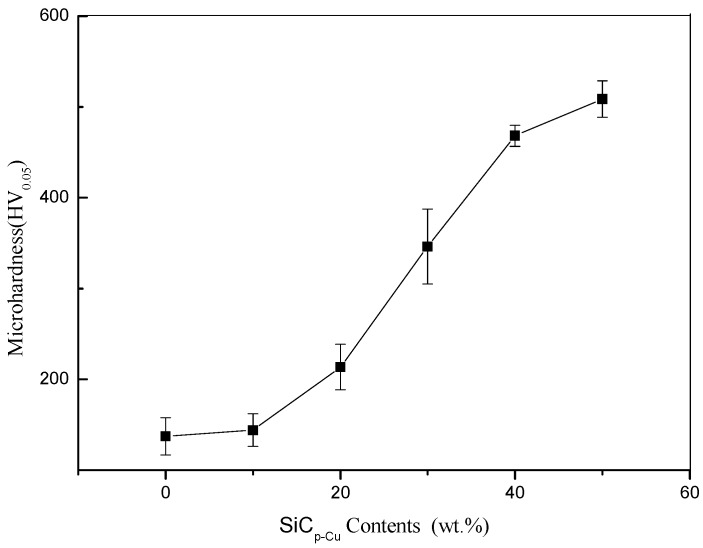
Relationship between microhardness and the SiC_p-Cu_ content.

Figure 9 shows the relationship between microhardness of LCLs measured on the cross-section and the SiC_p-Cu_ content in cladding materials. It can be seen that the average microhardness increases with the increasing SiC_p-Cu_ content. It is noteworthy that the average microhardness of the LCL with SiC_p-Cu_ content of 50 wt.% (508 HV_0.05_) is about 3.5 times higher than in the 4032 aluminum alloy substrate (145 HV_0.05_). 

**Figure 10:**
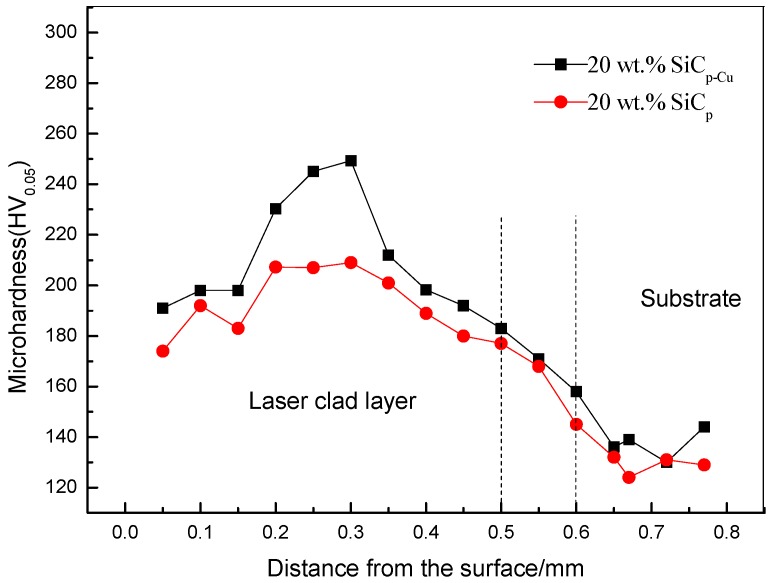
Distribution curves of microhardness along depth direction of SiC_p_/Al–Si laser cladding layer.

The variation of microhardness along depth direction of the LCLs is shown in Figure 10. As can be seen, the microhardness of LCL reinforced with SiC_p-Cu_ ranges from 190 HV_0.05_ to 250 HV_0.05_, and the average value is 210 HV_0.05_. The microhardness of the other LCL reinforced with SiC_p_ is between 170 and 209 HV_0.05_ and the average value is 192 HV_0.05_. This indicates that electroless copper plating on SiC_p_ can improve the microhardness of SiC_p_/Al–Si composite coating.

The two reasons for the high microhardness of the SiC_p_/Al–Si composite coating are as follows: Firstly, the increase in microhardness is mainly attributed to the dissolution of SiC_p-Cu_ in the LCLs and the resulting increase in the numbers of Al_4_SiC_4_ (1200 HV [30]) and Al–Cu intermetallic (microhardness of Al_2_Cu in the range of 400–600 HV_0.2_ [33]) formed on resolidification. Secondly, the effects of laser rapid heating and cooling cause a finer and harder microstructure.

### 3.4. Wear Properties

**Figure 11:**
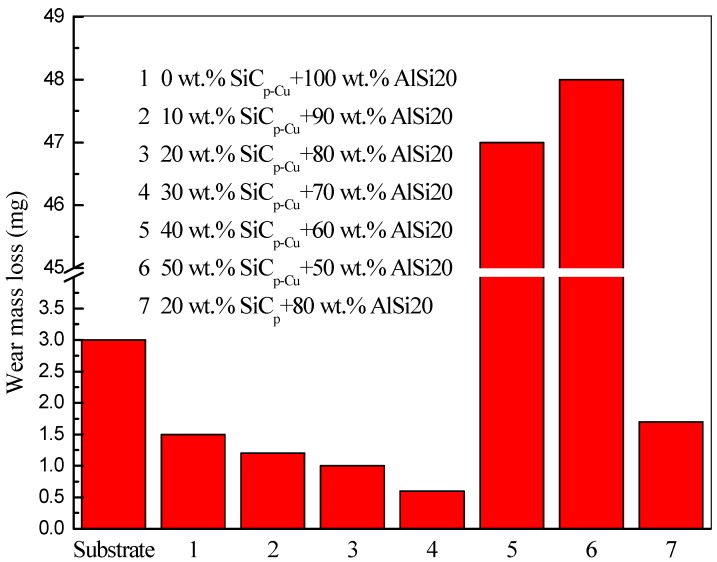
Wear mass loss of all samples.

**Figure 12:**
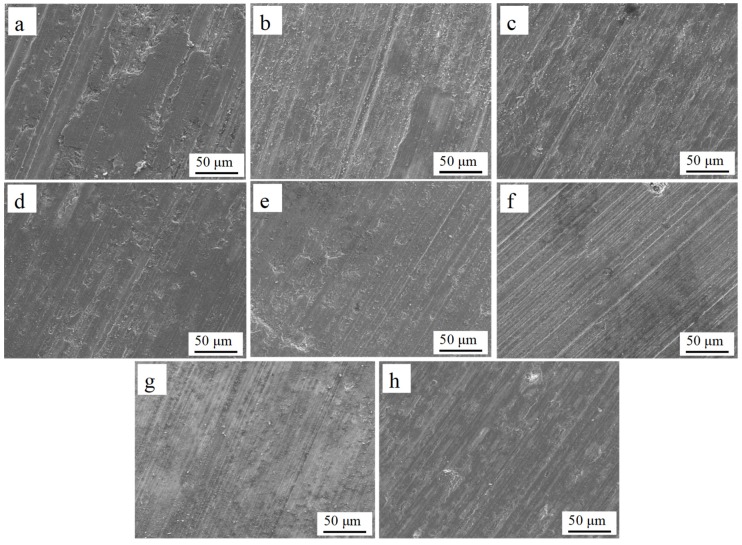
The worn surface morphologies of the substrate and laser cladding coatings with the different SiC_p_ contents; (**a**) substrate; (**b**) 100% AlSi20; (**c**) 10 wt.% SiC_p-Cu_ + 90 wt.% AlSi20; (**d**) 20 wt.% SiC_p-Cu_ + 80 wt.% AlSi20; (**e**) 30 wt.% SiC_p-Cu_ + 70 wt.% AlSi20; (**f**) 40 wt.% SiC_p-Cu_ + 60 wt.% AlSi20; (**g**) 50 wt.% SiC_p-Cu_ + 50 wt.% AlSi20; (**h**) 20 wt.% SiC_p_ + 80 wt.% AlSi20.

The wear mass loss of all samples is presented in Figure 11. It can be seen that the wear mass loss showed a decrease as the content of SiC_p-Cu_ increased from 0 to 30 wt.% and was less than that of the substrate. On the contrary, the LCLs with 40–50 wt.% of SiC_p-Cu_ had more wear mass loss compared with the substrate. The wear mass loss of LCL with a SiC_p_ content of 20 wt.% was less than that of the substrate, but more than that of the LCL with a SiC_p-Cu_ content of 20 wt.%. Figure 12 shows the SEM images of the worn surface of all samples. In Figure 12a, it is clearly observed that the worn surface of the substrate was easily deformed plastically under stress and shows parallel grooves. The grooves on the worn surface of the LCLs became narrower and shallower as the content of SiC_p-Cu_ increased from 0 to 30 wt.%, as shown in Figure 12b–e. The grooves on the worn surface of the LCL with 20 wt.% of SiC_p_ were deeper and wider compared with those of the LCL with 20 wt.% of SiC_p-Cu_. Nevertheless, it can be seen from Figure 12f–g that the grooves became deeper as the content of SiC_p-Cu_ increased from 40 to 50 wt.%. Therefore, the LCL with 30 wt.% of SiC_p-Cu_ exhibits the best wear resistance, and electroless copper plating on SiC_p_ can improve the wear resistance of SiC_p_/Al–Si composite coating. Improvement of wear resistance of the SiC_p_/Al–Si composite coating must be attributed to the presence of Al_4_SiC_4_ and Al–Cu intermetallic and finer microstructure. When the content of SiC_p-Cu_ reaches 40 wt.% or more, the content of AlSi20 decreases evidently, resulting in the difficulty of SiC_p-Cu_ packed by Al–Si alloy. The SiC_p-Cu_ were easily separated from the worn surface during the wear test and tended to plow the LCLs seriously. Consequently, the decrease of the wear resistance of the LCLs with high SiC_p-Cu_ content is observed.

## 4. Conclusions

1) SiC_p_-reinforced aluminum matrix composite coatings with high microhardness can be successfully obtained on the surface of 4032 aluminum alloy by the laser cladding process. Electroless copper plating on SiC_p_ can improve the properties of SiC_p_/Al–Si composite coating.
2) The microstructure of the SiC_p-Cu_/Al–Si laser cladding layer consisted of undissolved SiC_p_, lump-like primary Si, lump-like Al_2_Cu, plate-like Al_4_SiC_4_, and Al–Si–Cu ternary eutectic. Meanwhile, the microstructure became finer with the increasing of SiC_p-Cu_ content due to the fast solidification.
3) The microhardness of the laser cladding layer increased with the increasing of SiC_p-Cu_ content. It increased from 145 HV_0.05_ to 508 HV_0.05_ due to the presence of Al_4_SiC_4_ and Al–Cu intermetallic and finer microstructure.
4) The wear resistance of the laser cladding layer increased with the increasing of SiC_p-Cu_ content. The LCL reinforced with a SiC_p-Cu_ content of 30 wt.% exhibits the best wear resistance. When the SiC_p-Cu_ content reached 40-50 wt.%, the wear resistance of the LCLs decreased due to the spalling of SiC_p-Cu_ during the wear test.

## Figures and Tables

**Table 1:** Composition ratio of laser cladding material.

Number	SiC_p-Cu_ (wt.%)	SiC_p_ (wt.%)	Al (wt.%)
1	0	/	100
2	10	/	90
3	20	/	80
4	30	/	70
5	40	/	60
6	50	/	50
7	/	20	80

**Table 2:** Composition and operating conditions of pretreatment solutions and electroless copper plating bath.

	Roughening Solution	Sensitizing Solution	Activating Solution	Plating Bath
HF (40%)	1.15 M	/	/	/
HCl (37%)	/	1.20 M	0.24 M	/
SnCl_2_ · 2H_2_O	/	0.22 M	/	/
PdCl_2_	/	/	2.82 mM	/
CuSO_4_ · 5H_2_O	/	/	/	40 mM
NiSO_4_ · 6H_2_O	/	/	/	5.42 mM
NaH_2_PO_4_ · H_2_O	/	/	/	0.38 M
Na_3_C_6_H_5_O_7_ · 2H_2_O	/	/	/	0.14 M
H_3_BO_3_	/	/	/	0.48 M
T (°C)	25~30	25~30	25~30	65
pH	/	/	/	10.5
t (min)	15	15	15	10

**Table 3:** Intensity variation of Al diffraction peak of laser cladding with different SiC_p-Cu_ mass fraction.

SiC_p-Cu_ (wt.%)	2*θ* (°)	Intensity	FWHM	2*θ* (°)	Intensity	FWHM
standard	38.47			44.72		
10	38.49	1080	0.240	44.77	1817	0.262
20	38.56	1027	0.250	44.81	1005	0.283
30	38.53	1134	0.262	44.76	1467	0.284
40	38.48	449	0.294	44.74	226	0.356
50	38.41	630	0.252	44.66	326	0.304

**Table 4:** EDS analysis of laser cladding layer with a SiC_p-Cu_ content of 50 wt.% (corresponding to a, b, c, d points in Figure 6).

Detection Positions	Al (wt.%)	Si (wt.%)	C (wt.%)	Cu (wt.%)
a	26.03	50.01	2.40	21.56
b	45.01	10.80	3.87	40.32
c	32.30	11.15	36.94	20.62
d	55.74	4.09	2.37	37.81

## References

[1] Chi Y.M., Gu G.C., Yua H.J., Chen C.Z. (2018). Laser surface alloying on aluminum and its alloys: A review. Opt. Laser Eng..

[2] Jia Z.W., Sun W.C., Guo F., Dong Y.R., Liu X.J. (2018). Microstructure, friction and corrosion resistance properties of a Ni–Co–Al_2_O_3_ composite coating. RSC Adv..

[3] Watkins K.G., McMahon M.A., Steen W.M. (1997). Microstructure and corrosion properties of laser surface processed aluminium alloys: A review. Mater. Sci. Eng..

[4] Kawalec M., Przestacki D., Bartkowiak K., Jankowiak M. (2008). Laser assisted machining of aluminium composite reinforced by SiC particle. Int. Congr. Appl. Lasers Electro Opt..

[5] Wang C.L., Gao Y., Zeng Z.C., Fu Y.K. (2017). Effect of rare-earth on friction and wear properties of laser cladding Ni-based coatings on 6063Al. J. Alloy. Compd..

[6] Anandkumar R., Almeida A., Vilar R., Ocelík V., de Hosson J.T.M. (2009). Influence of powder particle injection velocity on the microstructure of Al–12Si/SiC_p_ coatings produced by laser cladding. Surf. Coat. Technol..

[7] Singh A., Ramakrishnan A., Baker D., Biswas A., Dinda G.P. (2017). Laser metal deposition of nickel coated Al 7050 alloy. J. Alloy. Compd..

[8] Xu N., Sarkar D.K., Chen X.G., Zhang H., Tong W.P. (2016). Superhydrophobic copper stearate/copper oxide thin films by a simple one-step electrochemical process and their corrosion resistance properties. RSC Adv..

[9] Hamid Z.A., Elkhair M.T.A. (2002). Development of electroless nickel–phosphorous composite deposits for wear resistance of 6061 aluminum alloy. Mater. Lett..

[10] Bao Y.Q., Gawne D.T., Gao J., Zhang T., Cuenca B.D., Alberdi A. (2013). Thermal-spray deposition of enamel on aluminium alloys. Surf. Coat. Technol..

[11] Lu J.Q., Wei G.Y., Yu Y.D., Guo C.F., Li J. (2018). Aluminum alloy AA2024 anodized from the mixed acid system with enhanced mechanical properties. Surf. Interface Anal..

[12] Liu W.H., Liu W.B., Bao A.L. (2012). Microstructure and Properties of Ceramic Coatings on 7N01 Aluminum Alloy by micro-Arc Oxidation. Procedia Eng..

[13] Riquelme A., Rodrigo P., Otero E., Rams J. (2017). Effect of alloy elements added on microstructure and hardening of Al/SiC laser clad coatings. J. Alloy. Compd..

[14] Riquelme A., Rodrigo P., Rams J. (2016). Analysis and optimization of process parameters in Al-SiC_p_ laser cladding. Opt. Laser. Eng..

[15] Podlesak H., Schnick T., Pawlowski L., Steinhäuser S., Wielage B. (2000). Microscopic study of Al–SiC particulate composites processed by laser shocks. Surf. Coat. Technol..

[16] Lee H.K. (2008). Effects of the cladding parameters on the deposition efficiency in pulsed Nd:YAG laser cladding. J. Mater. Process. Technol..

[17] Sun R.L., Lei Y.W. (2008). Microstructure and hardness of laser clad SiC_p_–Al composite coatings on Al alloys. Mater. Lett..

[18] Hegge H.J., Boetje J., de Hossonc J.T.H.M. (1990). Oxidation effects during laser cladding of aluminium with SiC/Al powders. J. Mater. Sci..

[19] Anandkumar R., Almeida A., Colaço R., Vilar R., de Hosson J.T.M. (2007). Microstructure and wear studies of laser clad Al-Si/SiC_(p)_ composite coatings. Surf. Coat. Technol..

[20] Viala J.C., Fortier P., Bouix J. (1990). Stable and metastable phase equilibria in the chemical interaction between aluminium and silicon carbide. J. Mater. Sci..

[21] Oden L.L., Mccune R.A. (1987). Phase equilibria in the Al-Si-C system. Metall. Trans. B.

[22] Zhou X.B., de Hosson J.T.M. (1995). Reactive wetting of liquid metals on ceramic substrates. Acta Mater..

[23] Ureña A., Rodrigo P., Gil L., Escalera M.D., Baldonedo J.L. (2001). Interfacial reactions in an Al-Cu-Mg (2009)/SiC_w_ composite during liquid processing Part II Arc welding. J. Mater. Sci..

[24] Chen Y.J., Cao M.S., Xu Q., Zhu J. (2003). Electroless nickel plating on silicon carbide nanoparticles. Surf. Coat. Technol..

[25] Zhu S.L., Tang L., Cui Z.D., Wei Q., Yang X.J. (2011). Preparation of copper-coated β-SiC nanoparticles by electroless plating. Surf. Coat. Technol..

[26] Chang F., Gu D.D., Dai D.H., Yuan P.P. (2015). Selective laser melting of in-situ Al_4_SiC_4_ + SiC hybrid reinforced Al matrix composites: Influence of starting SiC particle size. Surf. Coat. Technol..

[27] de Hosson J.T.M., Noordhuis J. (1994). Mechanical properties and microstructure of laser treated Al-Cu-Mg alloys. Mater. Sci. Forum.

[28] Guo H., Han Y.Y., Zhang X.M., Jia C.C., Xu J. (2015). Microstructure and thermophysical properties of SiC/Al composites mixed with diamond. Trans. Nonferrous Met. Soc. China.

[29] van Otterloo J.L.d.M., de Hosson J.T.M. (1994). Laser treatment of aluminum/copper alloys: A mechanical enhancement. Scripta Metall..

[30] de Wilde J., Froyen L. (2006). Microstructures observed during directional solidification along the univariant eutectic reaction in a ternary Al-Cu-Si alloy. Mater. Sci. Forum.

[31] Ferreira I.L., Moutinho D.J., Gomes L.G., Rocha O.L., Goulart P.R., Garcia A. (2010). Microstructural Development in a Ternary Al-Cu-Si Alloy during Transient Solidification. Mater. Sci. Forum.

[32] Agarwal A., Dahotre N.B. (1999). Laser surface engineering of steel for hard refractory ceramic composite coating. Int. J. Refract. Met. Hard Mater..

[33] Dubourg L., Pelletier H., Vaissiere D., Hlawka F., Cornet A. (2002). Mechanical characterisation of laser surface alloyed aluminium-copper systems. Wear.

